# Effect of Continuous Ingestion of 2 g of Medium-Chain Triglycerides on Substrate Metabolism during Low-Intensity Physical Activity

**DOI:** 10.3390/nu14030536

**Published:** 2022-01-26

**Authors:** Shougo Tsujino, Naohisa Nosaka, Shohei Sadamitsu, Kazuhiko Kato

**Affiliations:** 1 Central Research Laboratory, The Nisshin OilliO Group, Ltd., Kanagawa 235-8558, Japan; n-nosaka@nisshin-oillio.com (N.N.); s-sadamitsu@nisshin-oillio.com (S.S.); 2 Kato Clinic, 1-1-1 Nakaizumi, Komae, Tokyo 201-0012, Japan; kato-kaz@db3.so-net.ne.jp

**Keywords:** MCTs, fat oxidation, energy metabolism, low-intensity physical activity, obesity, respiratory exchange ratio

## Abstract

Increasing fat burning during physical activity is thought to be an effective strategy for maintaining health and preventing lifestyle-related diseases, such as obesity and diabetes. In recent years, medium-chain triglycerides (MCTs) have gained attention as a dietary component for increasing fat-burning. However, this fat-burning effect has been unclear in people with high body mass index (BMI). Therefore, we aimed to clarify the effects of 2 g of daily ingestion of MCTs over 2 weeks on substrate oxidation during low-intensity physical activity in sedentary (i.e., with no exercise habit) subjects with a BMI from 25 (kg/m^2^) to less than 30, which is classified as obese in Japan. A placebo-controlled, randomized, double-blind, crossover study with a 2-week washout period was conducted. The rate of fat oxidation as well as the respiratory exchange ratio (RER) during exercise (with a cycle ergometer at a 20-watt load) were measured with a human calorimeter. MCTs ingestion significantly increased fat oxidation during physical activity and decreased RER compared to long-chain triglycerides ingestion. In conclusion, we suggest that daily ingestion of 2 g of MCTs for 2 weeks increases fat burning during daily physical activities in sedentary persons with a BMI ranging from 25 to less than 30.

## 1. Introduction

Despite obesity being known to be a possible cause of lifestyle-related diseases such as diabetes and dyslipidemia, the number of obese Japanese people continues to increase due to changes in dietary habits and decreased physical activity resulting from changes in the living environment [1]. Since obesity is caused by an imbalance between energy expenditure and energy ingestion, preventing obesity by increasing physical activity and improving dietary habits (to ensure proper energy balance) are essential. While proper dietary energy intake is important, the use of fat as an energy substrate is also known to contribute significantly to obesity prevention, and various dietary components have been investigated for their effects on fat burning. For example, fish oil reportedly increases fat burning in the liver and enhances energy expenditure [2]. Moreover, catechins and capsaicin reportedly increase energy expenditure and fat oxidation at rest as well as during exercise [3,4,5,6], and reduce body fat [7,8].

Medium-chain fatty acids (MCFAs), which are components of coconut, palm fruit, and other palm plant seeds, are also found in human breast milk and cow’s milk [9]. MCFAs broadly refer to straight-chain saturated fatty acids consisting of 6–12 carbon chains, but research on physiological functions has focused on octanoic acid (C8) and decanoic acid (C10), or on medium-chain triglycerides (MCTs) consisting of C8 and C10. Continuous ingestion of MCTs has been reported to promote fat utilization during moderate-intensity exercise in recreational athletes [10] and during low-intensity daily activities in normal-weight subjects [11,12], and has been shown to reduce body weight and body fat [13,14].

In general, body mass index (BMI = weight (kg)/height (m)^2^) is the international standard used to determine obesity, and in Japan, obesity equates to a BMI of 25 (kg/m^2^) or higher [15]. The effect of continuous MCTs ingestion on fat oxidation during physical activity has been tested in normal weight subjects, but the effect on those with higher BMI is unknown. Although studies have been conducted with the ingestion dose of MCTs set at 6 g per day [11,12], if it can be shown that MCTs are effective even at lower doses then the burden of continuous ingestion will be reduced.

Therefore, the purpose of this study was to determine the effect of 2 g of daily ingestion of MCTs for 2 weeks on energy metabolism, especially fat oxidation, during low-intensity physical activity in people with a BMI from 25 to less than 30.

## 2. Materials and Methods

### 2.1. Study Conduct System and Ethical Considerations

This study was conducted in accordance with the Declaration of Helsinki, the Ethical Guidelines for Medical and Biological Research Involving Human Subjects [16], and the Act on the Protection of Personal Information [17]. The study was conducted by a contract research organization (CRO, Huma R&D Co., Ltd., Tokyo, Japan) under the guidance of a medical doctor at the Kouwa Clinic, Kouwa-kai Medical Corporation (Tokyo, Japan), and Fuji Medical Science Co., Ltd., Chiba, Japan. The study was reviewed and approved by the ethics committees of Yoga Allergy Clinic (approval number: 21000023, approval date: 12 February 2021). In addition, the contents of the study were registered in UMIN-CTR before the start of the study (UMIN-ID: UMIN000043022. Available online: https://upload.umin.ac.jp/cgi-open-bin/ctr/ctr.cgi?function=brows&action=brows&recptno=R000049074&type=summary&language=J) (accessed on 24 January 2022.

### 2.2. Subjects

Subjects were screened from the registration bank of the CRO after interviews, physical measurements, vital signs, stepping exercise and electrocardiography, biochemical tests, and hematological tests were performed, and those who met the following selection criteria from the viewpoint of human rights protection—i.e., did not violate the exclusion criteria, were able to comply with the administrative matters during the study period, and were judged by the investigator to have no problem participating in the study—were selected as subjects based on a comprehensive evaluation.

#### 2.2.1. Selection Criteria

(1) Japanese males and females aged at least 35 years and under 65 years at the time of obtaining written consent. (2) Those with a BMI from 25 to less than 30. (3) Non-smokers. (4) Those with an alcohol intake of less than 30 g/day. (5) Those who received a sufficient explanation of the purpose and content of the research, had the capacity to consent, had volunteered willingly to participate in the study, had a good understanding of the study, and agreed to participate in writing.

#### 2.2.2. Exclusion criteria

(1) Those who were currently receiving medical treatment or outpatient treatment for a serious disease. (2) Those who had experienced chest pain or abnormal pulse while at rest. (3) Those who were currently undergoing exercise or diet therapy under the medical supervision of a physician. (4) Those currently with a serious illness or complications, or a history of cardiac, hepatic, renal, cardiovascular, or hematological diseases. (5) Those who were undergoing treatment for rheumatism. (6) Those who frequently experienced shortness of breath, dizziness, vertigo, or loss of consciousness. (7) Those who were allergic to drugs, food, raw materials of test foods (soybeans, milk protein), or had a history of allergy. (8) Those who had a family member who had died suddenly of unknown causes. (9) Those who had been diagnosed as having disabilities in their legs and feet. (10) Those who feel pain in their hips, knees, or body when ascending or descending stairs. (11) Those who had knee surgery or disease or use a walking cane on a daily basis. (12) Subjects with a current or past history of drug or alcohol dependence. (13) Subjects with mental disorders (such as depression), sleep disorders (such as insomnia or sleep apnea), or those with a history of mental disorders in the past. (14) Those who exercise for the purpose of maintaining or improving their physical fitness for a total of 60 min or more per week. (15) Those who perform physical labor for a total of 10 h or more per week. (16) Those whose working hours were irregular due to night shifts. (17) Those with a weight fluctuation of more than ±5 kg within 2 months. (18) Those with extremely irregular lifestyles (including eating or sleeping). (19) Those who are extremely fussy eaters. (20) Those who take any health foods, supplements, or drugs that may affect fatigue reduction, fat burning, or obesity control. (21) Those who had participated in other clinical trials (research) within 3 months prior to the date of obtaining consent, or those who had plans to participate in other clinical trials (research) during the study period. (22) Those who had donated more than 200 mL of blood within 1 month (or 400 mL of blood within 3 months) prior to the date of consent. (23) Those who planned to donate blood or become vaccinated during the test period or those who wished to do so. (24) Those who were currently pregnant or breastfeeding, or who may become pregnant or breastfeed during the study period. (25) Those who had difficulty in complying with the recording on the various questionnaires. (26) Those who are judged to be unsuitable as subjects based on clinical laboratory values and measurements at the time of screening. (27) Any other person judged by the physician responsible for the study to be unsuitable as a subject.

### 2.3. Number of Target Subjects

Based on the mean difference and standard deviation of the cumulative fat oxidation and the maximum fat oxidation rate during low-intensity physical activity after ingestion of 6 g of MCTs for 2 weeks compared with the control [11], the number of subjects required for the study was estimated to be 18 or more and 23 or more. While the ingested amount of MCTs was one-third of that in the estimated study, a highly sensitive mass spectrometer was used to measure oxygen uptake and carbon dioxide production, which are the basic values for calculating fat oxidation, so the required number of subjects was judged to be slightly higher than the estimated number, 27 subjects were set, and so the target number of subjects was set at 30, based on the expectation that 10% of the subjects would be ineligible for analysis.

### 2.4. Test Foods and Dietary Surveys

MCTs (The Nisshin OilliO Group, Ltd., Tokyo, Japan) were used as the test diet, and long-chain triglycerides (LCTs) (The Nisshin OilliO Group, Ltd., Tokyo, Japan) were used as the control diet. The analyzed values of fatty acids’ composition of each oil are shown in Table 1.

Subjects were made to record their dietary status for 3 days prior to each test day. All meals were photographed with a camera, and the food was weighed (as far as possible) and nutritional information on the food was recorded. Nutritional values were calculated by a nutritionist and aggregate data was obtained. For the nutritional value calculations, the Japan Standard Tables of Food Composition 2015 (7th revision) were used [18].

### 2.5. Study Design

The study was conducted as a randomized, double-blind, crossover trial with the control diet being the placebo. A third party not involved in the study (the person responsible for the allocation of the test foods) randomly assigned the subjects to two groups: a group that ingested the control diet first and a group that ingested the test diet first. After assignment, the person responsible for the allocation of the test foods confirmed that there were no significant differences in gender and age. Moreover, this person kept the test foods allocation list strictly to themselves until this study finished, and blinding was maintained for all parties except the person responsible for the allocation of the test foods.

**Figure 1:**
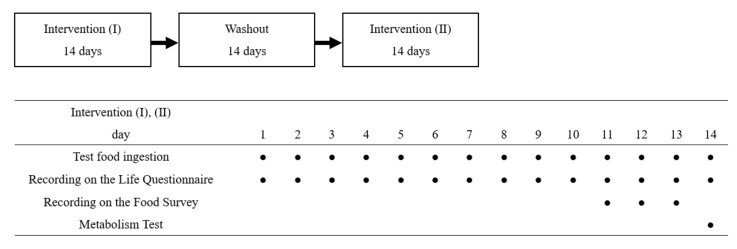
Study design. This study was conducted as a crossover study. There was a 14-day washout period between intervention periods I and II. During the intervention period, the subjects were asked to ingest the test food every day and to record their living conditions on the Life Questionnaire. For three days prior to each test day, the subjects were asked to take pictures of all their meals with a camera, and in addition they were asked to measure as much food as possible and take pictures of food labels to record their dietary status. On day 14 of each intervention period, a metabolic test was conducted.

The test food (test diet (MCTs) or control diet (LCTs)) was ingested by the subjects at a rate of 2 g per day from 13 days before the day of the metabolic test. On the day of the test, the subjects were asked to ingest the assessed food comprising 483 kcal of energy, 15.5 g of protein, 15.2 g of fat, and 75.1 g of carbohydrate, including the test food at the metabolic test site. The study design is shown in Figure 1.

Subjects were instructed to keep their body weight as unchanged as possible and observe the following guidelines during the study period.

#### 2.5.1. Compliance Issues during the Study Period

(1) During the study period, the subjects were told to maintain a normal lifestyle and to limit any excessive exercise, overeating, and heavy drinking that significantly deviated from their daily lives. (2) During the study period, subjects were also told to make as few changes as possible to their lifestyle and environment—such as meals, alcohol consumption (less than 30 g/day), exercise, sleep, and work—prior to their participation in the study. (3) During the study period, those with exercise habits were told not to engage in more strenuous exercise than usual and not to change the quantity or quality of their exercise. Those who were sedentary were told not to start a new exercise program. (4) Subjects were instructed not to ingest any medicines, supplements, health foods, or functional foods that might affect the reduction of fatigue, fat burning, or obesity during the study. If a new ingestion was unavoidable and became necessary, then they were told to contact the CRO in advance whenever possible. If they did ingest such items, they had to record the details on the Lifestyle Questionnaire. (5) During the study period, the status of the subjects ingesting any previously habitual and unrestricted medicines, supplements, health foods, or functional foods had to be recorded on the Lifestyle Questionnaire, and the subjects did not change their habits. In addition, those who did not have a habit were told that they should not start a new ingestion. If a new ingestion became necessary due to unavoidable circumstances, then the subject needed to contact CRO as far in advance as possible. (6) During the study period, the subjects were told not to ingest oils for health (such as coconut oil or flaxseed oil) that might enhance the dietary components being evaluated. (7) During the study period, subjects could continue to ingest milk, butter, ice cream, and other foods rich in milk fat as usual, but were not to significantly change the amount or frequency of their ingestion. If there was any change, it had to be recorded on the Lifestyle Questionnaire. (8) During the study period, the subjects were told not to participate in other clinical trials (research). (9) During the study period, subjects could not participate in whole blood donation or component blood donation. (10) During the study period, those who wished to undergo a medical checkup or physical examination had to report to the CRO in advance and record the information on the Lifestyle Questionnaire. (11) The subjects were told not to divulge any information about the content of the study or information obtained through participation in the study to others. (12) Test foods were ingested as instructed by the CRO. (13) During the designated period, the subjects had to record their living conditions on a daily life survey form using a system that allowed them to write a diary on an electronic terminal. (14) If subjects had any physical problems during or after the study, then they had to contact the CRO immediately and record the details on the Lifestyle Questionnaire.

#### 2.5.2. Compliance Matters before Metabolic Test

(1) Subjects had to refrain from strenuous exercise during the 3 days leading up to the test. (2) Subjects could not drink alcohol on the day before the test. (3) The day before the test, they were to keep to the same time and quality of sleep as much as possible during intervention period I and II. (4) Subjects were told that they should remain in a fasting state (drinking water only) from 9:00 p.m. on the day prior to the test until the designated time for ingestion of the food to be evaluated. In addition, in the last meal or drink before fasting, fatty foods and large amounts of sweetened beverages were to be avoided. (5) They were to record their diet for 3 days prior to the test.

#### 2.5.3. Compliance Matters on the Day of the Metabolic Test

(1) On the day of the metabolic test, subjects were to refrain from eating breakfast and had to ingest the assessed food at the designated time. (2) If subjects were unable to come to the test site for any reason on the day of the test, they were required to contact the CRO immediately.

### 2.6. Metabolism Measurements

For the metabolic tests in this study, metabolic measurements were conducted using a human calorimeter (Fuji Human Calorimeter FHC-30S, Fuji Medical Science Co., Ltd., Chiba, Japan). The internal dimensions of the normal pressure type human calorimeter were 3.80 m × 3.00 m × 2.40 m, and the volume of the chamber was 27.36 m^3^. The chamber was equipped with a desk, chair, toilet, TV, and cycle ergometer (STB-3400, Nihon Kohden Corporation, Tokyo, Japan). The inflow rate of air into the chamber was set to 50 L/min, and the air supply and exhaust were controlled at the same volume with an accuracy of ±0.5%. The temperature and humidity of the chamber were controlled at 25 °C ± 0.1 °C and 50% ± 1.0%, respectively. The rate of oxygen uptake and rate of carbon dioxide production were analyzed using Brown’s equation [19] and obtained every minute, based on the oxygen concentration, carbon dioxide concentration, air supply volume, and exhaust volume measured by the human calorimeter. The accuracy of the analyzer for the exhausted gas was ±0.002%.

In order to examine the accuracy of the measurement during this study, alcohol combustion tests were conducted before, during, and after the metabolic test. For the oxygen uptake rate and the carbon dioxide production rate, the theoretical values were calculated from the weight of alcohol burned, and the measured values were calculated by analyzing the data obtained from the measurements using Henning’s equation [20]. The recovery rate, which represents the measurement accuracy, was calculated from the theoretical and measured values (formula: (measured value ÷ theoretical value) × 100; unit: %), and the recovery rates of the oxygen uptake rate and carbon dioxide production rate were both within 100 ± 2%.

Eighty minutes before the ingestion of the assessed food, the subjects entered the human calorimeter. The subjects maintained a sitting rest in the chamber and measured their resting metabolism before ingesting the assessed food. After the ingestion of the assessed food, the subjects were again kept in a sitting position in the chamber. Four hours after the ingestion of the assessed food, their resting metabolism was measured, and after confirming that the energy expenditure rate equated to that before the ingestion of the assessed food, the metabolism during physical activity was measured. Metabolism during physical activity was measured using a cycle ergometer with a 20-watt load, and the subjects were asked to maintain the speed at 50 revolutions per minute for 30 min. Among the metabolic data during the 30-min physical activity, the data from 2 to 30 min after the start of physical activity (which are considered to reflect the metabolic increase) were adopted.

The fat oxidation rate, carbohydrate oxidation rate, respiratory exchange ratio, and energy expenditure rate in physical activity metabolism were calculated from the oxygen uptake rate and carbon dioxide production rate determined by Brown’s equation, using the following equation based on previous reports [21].

- Fat oxidation rate: 1.689 × oxygen uptake rate–1.689 × carbon dioxide production rate.
- Carbohydrate oxidation rate: 4.113 × carbon dioxide production rate–2.907 × oxygen uptake rate.
- Respiratory exchange ratio: carbon dioxide production rate ÷ oxygen uptake rate.
- Energy expenditure rate: 3.941 × oxygen uptake rate + 1.106 x carbon dioxide production rate.

### 2.7. Diagnosis

During the study period, adverse events were investigated by daily Lifestyle Questionnaire. In the event of an adverse event, the physician responsible for the study would take necessary and appropriate action immediately, evaluate the adverse event, and make a judgment on six levels: “none”, “probably none”, “maybe”, “probably yes”, “yes”, and “unevaluable”. Adverse events (other than those judged by the physician responsible for the study to be “probably none” or “none” causally related to the test food) were considered as adverse reactions.

### 2.8. Primary and Secondary Outcomes

The primary outcomes were fat oxidation rate and respiratory exchange ratio. Secondary outcomes were energy expenditure rate, carbohydrate oxidation rate, and maximum fat oxidation rate.

### 2.9. Statistical Analysis

Subject background and nutrient intake are shown as mean ± standard deviation. For the measurement outcomes, values of the mean difference between values in the test and control diet groups (intervention effect values) were calculated and shown as mean ± standard deviation.

Nutrient intake during the intervention period was compared between groups and checked for normality by Shapiro-Wilk test. If the Shapiro-Wilk test showed significance, the Mann-Whitney U test was conducted, if not significant the F test was used to check for equal variance, if the F test was not significant the Student’s t test was conducted, and if significant the Welch’s t test was conducted. Mann-Whitney U test was performed for each measurement item in physical activity metabolism.

The validity of the crossover method was verified by testing the carryover effect of repetition using a linear model for each measurement item for which a significant difference was found.

Basic statistics of the analyzed data were calculated using Microsoft Excel for Office365 MSO (Microsoft Japan Corporation, Tokyo, Japan), and R statistical software, v4.1.0 for Windows (R Core Team, Vienna, Austria) was used for statistical processing. In all significant difference analyses, a risk ratio of less than 5% was considered significant, and a risk ratio of 5–10% was considered a tendency.

## 3. Results

### 3.1. Analysis of Subjects

**Figure 2:**
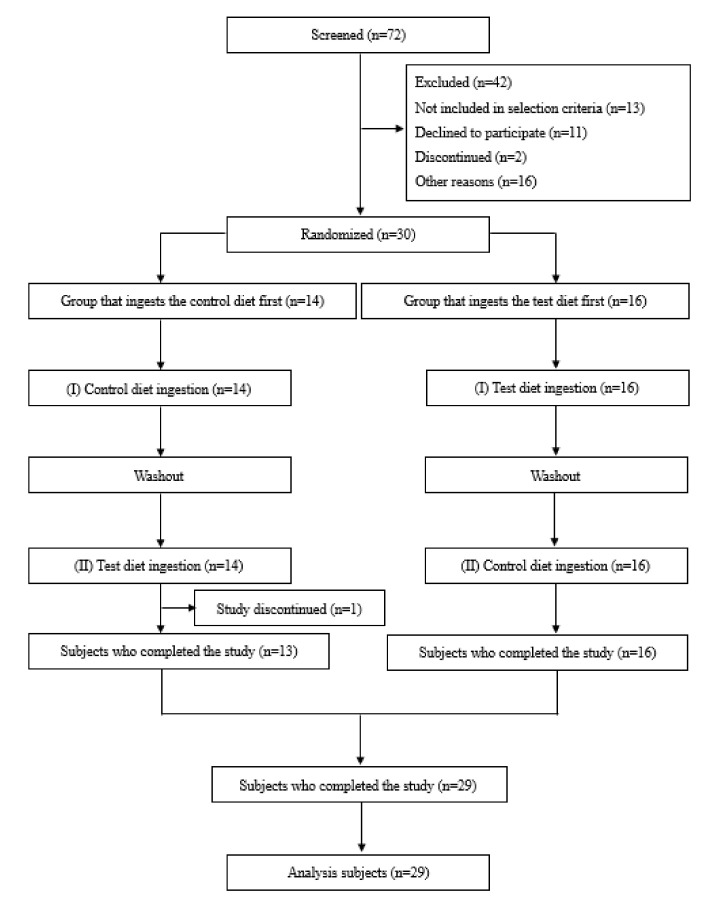
Flowchart of study subjects. 72 candidates were screened (SCR) by interview, physical measurements, vital signs, stepping exercise and electrocardiography, biochemical and hematological tests. 42 candidates were excluded and 30 subjects were included in the study. 30 subjects were randomly assigned to two groups: one group (14 subjects) ingested the control diet first, and the other group (16 subjects) ingested the test diet first. One subject discontinued the study before the second metabolic test, and the total number of completed subjects was 29, who were included in the analysis.

From 72 potential subjects who consented, 30 subjects who met the selection criteria and did not violate the exclusion criteria were included in the study at the discretion of their physicians. Subsequently, one subject withdrew (because of ill health due to tonsillitis) before the metabolic test in intervention period II, leaving 29 subjects who completed the study. These 29 subjects were used as the subjects for analysis (Figure 2). The background information for the analyzed subjects is shown in Table 2.

### 3.2. Adverse Events

30 subjects were reviewed for adverse events and evaluated by the physician responsible for the study. Although 24 adverse events were observed during the study period, all events were judged by the responsible physician to be “not related” to the test food, and no adverse events were attributed to the test food.

### 3.3. Nutrient Intake

The average daily nutrient intake for the 3 days prior to each test day is shown in Table 3. Octanoic acid intake and decanoic acid intake were significantly higher in the test diet group than in the control diet group. As a result, saturated fatty acid intake was significantly higher in the test diet group than in the control diet group.

### 3.4. Test Results of Measurement Outcomes

The intervention effect values for each measurement outcome are shown in Table 4.

#### 3.4.1. Primary Measurement Outcomes:

The intervention effect value of fat oxidation rate was 8.8 ± 28.6 mg/min, which was significantly higher in the test diet group. The intervention effect value of respiratory exchange ratio was −0.01 ± 0.03, which was significantly lower in the test diet group.

##### 3.4.2. Secondary Measurement Outcomes:

The rate of energy expenditure was not significantly different between the two groups. The intervention effect value for carbohydrate oxidation rate was −18.7 ± 52.9 mg/min, which was significantly lower in the test diet group. The intervention effect value of the maximum fat oxidation rate was 11.1 ± 62.0 mg/min, which tended to be higher in the group consuming the test diet.

### 3.5. Results of the Test for the Carryover Effect of Repetition

A linear model was used to test the carryover effect of repetition for each of the endpoints that were found to be significant in the efficacy assessment. The *p*-values for fat oxidation ratio, respiratory exchange ratio, and carbohydrate oxidation ratio were 0.90, 0.60, and 0.59, respectively, indicating that there was no significant carryover effect of repetition for any of the items.

## 4. Discussion

The results of this study showed that continuous ingestion of MCTs significantly increased the fat oxidation rate and significantly decreased the respiratory exchange ratio during low-intensity physical activity in persons with a BMI from 25 to less than 30 and who were sedentary. In addition, compared to the control diet group, the energy expenditure rate during physical activity in the test diet group was not significant, and the carbohydrate oxidation rate was significantly decreased. These results indicate that MCTs enhance fat burning by preferentially utilizing fat as an energy substrate during low-intensity physical activity in those classified as obese by Japanese standards. Furthermore, the results showed that MCTs have an enhanced fat-burning effect when 2 g are ingested daily.

The mechanism by which continuous ingestion of MCTs increases fat oxidation during low-intensity physical activity may be due to an increase in the number (volume) of mitochondria and to increased fat catabolism in skeletal muscle. A mitochondrion is an intracellular organelle that oxidizes glucose and lipids to produce ATP, which is necessary for muscle contraction. Previous studies in animals have reported that continuous ingestion of MCTs increases mitochondrial biosynthesis [22] and activates metabolism-related enzymes to enhance their metabolism [22,23,24]. The Akt and AMPK signaling pathways are reportedly involved in these mitochondrial changes [22,24]. In addition, it has been shown that fat oxidation capacity is increased by increasing mitochondrial density in skeletal muscle [25] and, in a study that showed that continuous ingestion of MCTs improved endurance exercise capacity during swimming, the promotion of fat burning during exercise was shown to be important for improving endurance exercise capacity [23,26].

A high-fat diet, which is higher than the daily intake of LCTs, also reportedly increases the number of mitochondria and metabolism in skeletal muscle via PPARδ, resulting in increased fat burning [27,28,29]. However, there are many issues to be considered, including the fact that continuing a high-fat diet is detrimental because it involves a drastic change in diet, and even if it can be continued, it will increase body weight and body fat, which is concerning [30]. On the other hand, it has been shown that the application of 6 g of MCTs per day causes increased fat burning in skeletal muscle even in those on a normal fat diet, with or without exercise habits [10,11,12]. Furthermore, this study showed that a continuous ingestion of only 2 g of MCTs per day can enhance fat burning during low-intensity physical activity. Although it has been shown that MCTs are safe to ingest [31], it is known that diarrhea can occur when they are ingested in large amounts [32]. Therefore, we believe that it is significant that the results of this study demonstrated that MCTs function even when ingested in smaller amounts.

MCFAs (C8 and C10) are components found in human breast milk at about 1.5–2.9%, in dairy products at about 4.0–4.7%, and in coconut oil at about 13.9% of the fatty acid content [33]. Japanese people consume about 0.2 g to 0.3 g per day from dairy products [33]. Therefore, the present study indicates that continuous intake of C8 and C10, about 10 times higher than daily intake, may enhance fat burning more than daily intake of LCTs.

According to the “Physical Activity Standards for Health Promotion 2013 (“2013 Standards”)” compiled by [34] as part of the efforts to promote “Healthy Japan 21, 2nd stage” [35], physical activity refers to all activities that expend more energy than in a state of rest, and is divided into activities of daily living (“daily activities”) and exercise designed to maintain and improve physical fitness. The physical activity intensity in this study was converted into METs (metabolic equivalents), which averaged about 2.2 METs.

This physical activity intensity is equivalent to daily activities such as “doing the laundry, folding or hanging clothes, putting clothes in the washer or dryer, packing a suitcase, washing clothes by hand, implied standing, and light effort (2.0 METs)”; “cleaning, sweeping, or slow, light effort (2.3 METs)”; and “food shopping with or without a grocery cart, standing, or walking (2.3 METs)” [36]. In other words, continuous ingestion of 2 g of MCTs per day could enhance fat burning during activities of daily living. The 2013 standards show that increased physical activity and habitual aerobic exercise increase energy expenditure, utilize visceral and subcutaneous fat as an energy source, and reduce abdominal circumference and weight.

This study showed for the first time that continuous (daily) ingestion of 2 g of MCTs enhances fat burning during daily activities in sedentary people with high BMI. Previous studies in which humans ingested MCFAs equivalent to 2 g of MCTs showed an increase in postprandial thermogenesis during a single ingestion and a decrease in body weight and body fat during continuous ingestion [37,38]. We consider that the results of this study indicate that the anti-obesity effect of the ingestion of MCTs equivalent to 2 g is due to the enhancement of fat burning in daily activities during continuous ingestion, in addition to the effect of increased diet-induced thermogenesis after MCFAs ingestion.

In previous studies on the effects of MCFAs ingestion in animals, it was reported that MCFAs increased fat catabolism in skeletal muscle [39], liver [40], and adipose tissue [41], each of which are organs where fat catabolism is actively performed. We believe that these reports represent a more robust mechanism of action for the anti-obesity effects shown in human studies. The enhancement of fat burning during daily activities in sedentary people with a high BMI shown in this study is thought to contribute to the reduction of body fat through the continuous ingestion of MCFAs, and is expected to contribute to the prevention and reduction of obesity.

The present study was conducted under conditions without weight fluctuation. Therefore, no weight loss was observed after 2 weeks of MCTs ingestion. The energy intake during the intervention period was about 200 kcal more in the test diet group, although the difference was not statistically significant. This could be attributed to the fact that subjects ate more food in order to maintain their weight. Therefore, we believe that there is a possibility that weight change can be observed even after two weeks of MCT ingestion of 2 g per day. On the other hand, there was no difference in energy expenditure during physical activity, which is thought to lead to weight loss, between control diet group and test diet group. Further studies are needed to reveal the mechanism by which continuous MCTs ingestion reduces body weight. 

It is known that physical activity is related to life expectancy and disease risk [42,43], and increasing the amount of physical activity is an important factor in maintaining and improving health. Indeed, fat catabolism is enhanced by daily endurance exercise [44], and recently it has been shown that the amount of daily activity may contribute to fat oxidation capacity [45]. In addition, animal studies have shown that MCTs ingestion and exercise additively enhance metabolic rate and reduce visceral fat [46]. Hence, considering these points, we believe that combining MCTs ingestion with daily activities can be expected to more strongly prevent or reduce obesity and contribute to extending healthy life expectancy. 

Recent changes in our “obesogenic” living environment, such as motorization and increased time spent sitting at home due to pandemic, have unintentionally reduced our physical activity, but we hope to encourage people to increase their physical activity based on the effects of continuous MCTs ingestion, shown in this study to increase fat burning during daily activities.

In this study, healthy sedentary men and women with higher than average BMI were tested under low-intensity physical activity load conditions at the daily activity level using a bicycle ergometer. There are some limitations associated with this study. (1) The effect on people with a BMI of less than 25 as well as exercise enthusiasts and athletes are unknown. (2) The effects of moderate-intensity and higher physical activity load conditions are unknown.

## 5. Conclusions

A placebo-controlled, randomized, double-blind, crossover study was conducted to evaluate the effects of daily ingestion of 2 g MCTs for 2 weeks on fat catabolism during low-intensity physical activity in sedentary individuals and with a BMI from 25 to less than 30. The results showed that MCTs ingestion significantly increased the rate of fat oxidation during low-intensity physical activity, indicating that it enhances fat catabolism. These results suggest that daily ingestion of 2 g of MCTs over 2 weeks increased fat burning during daily physical activities in sedentary persons with a BMI from 25 to less than 30. As a possible mechanism for the enhanced fat-burning effect observed with MCTs ingestion during daily activities, increased mitochondrial biosynthesis and enhanced mitochondrial metabolism were considered to be contributory factors. We have shown that continuous ingestion of MCTs in amounts more easily adapted to daily lifestyle enhances fat burning during daily activities in sedentary obese individuals, but further studies are needed to understand the mechanisms of weight loss.

## Figures and Tables

**Table 1:** Fatty acids’ composition (g/100 g total fatty acids). MCTs (The Nisshin OilliO Group, Ltd., Tokyo, Japan) were used as the test diet, and LCTs (The Nisshin OilliO Group, Ltd., Tokyo, Japan) were used as the control diet. The fatty acids’ composition analysis values of each oil are shown.

Fatty Acid ^1^	LCTs	MCTs
8:0	ND ^2^	74.4
10:0	ND	25.6
16:0	4.2	ND
18:0	2.0	ND
18:1	59.6	ND
18:2	20.4	ND
18:3	10.2	ND
Others	3.6	ND

^1^ Number of carbon atoms: Number of double bonds. ^2^ Not detected.

**Table 2:** Background information for the 29 subjects in the analysis ^1^.

Characteristics		*n* = 29
Age	years	50.3 ± 9.1
Sex	M/F	17/12
Height	cm	165.4 ± 8.9
Body weight	kg	73.9 ± 9.5
BMI	kg/m^2^	26.8 ± 1.3

^1^ Values are shown as mean ± standard deviation.

**Table 3:** Nutrient intakes during the intervention period ^1^. The average intake of nutrients during the three days prior to each metabolic test.

		*n* = 29	
		Control Diet	Test Diet
Energy	kcal	1798.2 ± 316.1	1974.8 ± 359.4
Protein	g	67.2 ± 14.2	71.3 ± 16.5
Fat	g	64.8 ± 15.6	73.7 ± 22.6
Saturated fatty acid	g	18.1 ± 5.6	22.4 ± 7.3 *
Octanoic acid	mg	95.9 ± 90.0	1969.4 ± 125.5 *
Decanoic acid	mg	188.2 ± 169.8	865.1 ± 200.5 *
Carbohydrate	g	226.5 ± 61.8	246.2 ± 57.3

^1^ Values are shown as mean ± standard deviation. * Significant difference compared to control group (*p* < 0.05).

**Table 4:** Metabolic data during physical activity ^1^. The energy expenditure rate, fat oxidation rate, carbohydrate oxidation rate, respiratory exchange ratio, and maximum fat oxidation rate during exercise in the metabolic test.

*n* = 29		Control Diet	Test Diet	Intervention Effect Value (Test Diet–Control Diet)	*p* Value
Energy expenditure rate	kcal/min	2.40 ± 0.24	2.41 ± 0.22	0.01 ± 0.14	0.82
Fat oxidation rate	mg/min	174.7 ± 46.2	183.4 ± 41.5	8.8 ± 28.6 *	0.01
Carbohydrate oxidation rate	mg/min	175.3 ± 76.5	156.6 ± 61.5	−18.7 ± 52.9 *	0.03
Respiratory exchange ratio		0.79 ± 0.04	0.79 ± 0.03	−0.01 ± 0.03 *	0.03
Maximum fat oxidation rate	mg/min	321.4 ± 66.0	332.6 ± 62.1	11.1 ± 62.0 ^†^	0.052

^1^ Values are shown as mean ± standard deviation. Significant difference compared to control group * (*p* < 0.05). There is a trend compared to the control group ^†^ (*p* < 0.1).

## Data Availability

Data not available due to commercial restrictions.

## References

[1] Ministry of Health, Labour and Welfare (2019). The National Health and Nutrition Survey in Japan.

[2] Ezaki O. (2006). Prevention of Lifestyle-related Disease by Regular Exercise and Fish Oil Feeding. J. Jpn. Soc. Nutr. Food Sci..

[3] Harada U., Chikama A., Saito S., Takase H., Nagao T., Hase T., Tokimitsu I. (2005). Effects of the Long-Term Ingestion of Tea Catechins on Energy Expenditure and Dietary Fat Oxidation in Healthy Subjects. J. Health Sci..

[4] Inoue N., Matsunaga Y., Satoh H., Takahashi M. (2007). Enhanced energy expenditure and fat oxidation in humans with high BMI scores by the ingestion of novel and non-pungent capsaicin analogues (capsinoids). Biosci. Biotechnol. Biochem..

[5] Ota N., Soga S., Shimotoyodome A., Haramizu S., Inaba M., Murase T., Tokimitsu I. (2005). Effects of Combination of Regular Exercise and Tea Catechins Intake on Energy Expenditure in Humans. J. Health Sci..

[6] Shin K.O., Moritani T. (2007). Alterations of autonomic nervous activity and energy metabolism by capsaicin ingestion during aerobic exercise in healthy men. J. Nutr. Sci. Vitaminol..

[7] Nagao T., Hase T., Tokimitsu I. (2007). A green tea extract high in catechins reduces body fat and cardiovascular risks in humans. Obesity.

[8] Snitker S., Fujishima Y., Shen H., Ott S., Pi-Sunyer X., Furuhata Y., Sato H., Takahashi M. (2009). Effects of novel capsinoid treatment on fatness and energy metabolism in humans: Possible pharmacogenetic implications. Am. J. Clin. Nutr..

[9] Babayan V.K. (1987). Medium chain triglycerides and structured lipids. Lipids.

[10] Nosaka N., Suzuki Y., Suemitsu H., Kasai M., Kato K., Taguchi M. (2018). Medium-chain Triglycerides with Maltodextrin Increase Fat Oxidation during Moderate-intensity Exercise and Extend the Duration of Subsequent High-intensity Exercise. J. Oleo Sci..

[11] Nosaka N., Tsujino S., Honda K., Suemitsu H., Kato K. (2020). Enhancement of Fat Oxidation during Submaximal Exercise in Sedentary Persons: Variations by Medium-Chain Fatty Acid Composition and Age Group. Lipids.

[12] Nosaka N., Tsujino S., Honda K., Suemitsu H., Kato K., Kondo K. (2021). Effect of ingestion of medium-chain triglycerides on substrate oxidation during aerobic exercise could depend on sex difference in middle-aged sedentary persons. Nutrients.

[13] Tsuji H., Kasai M., Takeuchi H., Nakamura M., Okazaki M., Kondo K. (2001). Dietary medium-chain triacylglycerols suppress accumulation of body fat in a double-blind, controlled trial in healthy men and women. J. Nutr..

[14] Nosaka N., Maki H., Suzuki Y., Haruna H., Ohara A., Kasai M., Tsuji H., Aoyama T., Okazaki M., Igarashi O. (2003). Effects of margarine containing medium-chain triacylglycerols on body fat reduction in humans. J. Atheroscler. Thromb..

[15] Japan Society for the Study of Obesity (2016). Guidelines for the Management of Obesity Disease.

[16] Ministry of Health, Labour and Welfare, Japan (2013). Ethical Guidelines for Medical and Biological Research Involving Human Subjects.

[17] Ministry of Justice, Japan (2003). Act on the Protection of Personal Information.

[18] Ministry of Education, Culture, Sports, Science and Technology, Japan (2015). Standard Tables of Food Composition in Japan.

[19] Brown D., Cole T.J., Dauncey M.J., Marrs R.W., Murgatroyd P.R. (1984). Analysis of gaseous exchange in open-circuit indirect calorimetry. Med. Biol. Eng. Comput..

[20] Henning B., Löfgren R., Sjöström L. (1996). Chamber for indirect calorimetry with improved transient response. Med. Biol. Eng. Comput..

[21] Nieman D.C., Simonson A., Sakaguchi C.A., Sha W., Blevins T., Hattabaugh J., Kohlmeier M. (2019). Acute Ingestion of a Mixed Flavonoid and Caffeine Supplement Increases Energy Expenditure and Fat Oxidation in Adult Women: A Randomized, Crossover Clinical Trial. Nutrients.

[22] Ishizawa R., Masuda K., Sakata S., Nakatani A. (2015). Effects of different fatty acid chain lengths on fatty acid oxidation-related protein expression levels in rat skeletal muscles. J. Oleo Sci..

[23] Fushiki T., Matsumoto K., Inoue K., Kawada T., Sugimoto E. (1995). Swimming endurance capacity of mice is increased by chronic consumption of medium-chain triglycerides. J. Nutr..

[24] Wang Y., Liu Z., Han Y., Xu J., Huang W., Li Z. (2018). Medium Chain Triglycerides enhances exercise endurance through the increased mitochondrial biogenesis and metabolism. PLoS ONE..

[25] Iossa S., Mollica M.P., Lionetti L., Crescenzo R., Botta M., Liverini G. (2002). Skeletal muscle oxidative capacity in rats fed high-fat diet. Int. J. Obes. Relat. Metab. Disord. Int. J. Obes..

[26] Fushiki T. (2000). Nutrition During Exercise and Training. Jpn. J. Nutr. Diet..

[27] Wang Y.-X., Lee C.-H., Tiep S., Yu R.T., Ham J., Kang H., Evans R.M. (2003). Peroxisome-proliferator-activated receptor delta activates fat metabolism to prevent obesity. Cell.

[28] Garcia-Roves P., Huss J.M., Han D.H., Hancock C.R., Iglesias-Gutierrez E., Chen M., Holloszy J.O. (2007). Raising plasma fatty acid concentration induces increased biogenesis of mitochondria in skeletal muscle. Proc. Natl. Acad. Sci. USA.

[29] Hancock C.R., Han D.H., Chen M., Terada S., Yasuda T., Wright D.C., Holloszy J.O. (2008). High-fat diets cause insulin resistance despite an increase in muscle mitochondria. Proc. Natl. Acad. Sci. USA.

[30] TERADA S. (2018). Lipid Nutrition: New insights into Sports Nutrition. Oleoscience.

[31] Traul K.A., Driedger A., Ingle D.L., Nakhasi D. (2000). Review of the toxicologic properties of medium-chain triglycerides. Food Chem. Toxicol..

[32] Ivy J.L., Costill D.L., Fink W.J., Maglischo E. (1980). Contribution of Medium and Long Chain Triglyceride Intake to Energy Metabolism During Prolonged Exercise. Int. J. Sports Med..

[33] Aoyama T. (2003). Nutritional Studies on Medium-Chain Fatty Acid—From the Recent Research. Oleoscience.

[34] Ministry of Health, Labour and Welfare, Japan (2013). Physical Activity Reference for Health Promotion 2013.

[35] Ministry of Health, Labour and Welfare, Japan (2013). Health Japan 21, the Second Term.

[36] Ainsworth B.E., Haskell W.L., Herrmann S.D., Meckes N., Bassett D.R., Tudor-Locke C., Greer J.L., Vezina J., Whitt-Glover M.C., Leon A.S. (2011). 2011 Compendium of Physical Activities: A second update of codes and MET values. Med. Sci. Sports Exerc..

[37] Kasai M., Nosaka N., Maki H., Negishi S., Aoyama T., Nakamura M., Suzuki Y., Tsuji H., Uto H., Okazaki M. (2003). Effect of dietary medium- and long-chain triacylglycerols (MLCT) on accumulation of body fat in healthy humans. Asia Pac. J. Clin. Nutr..

[38] Ogawa A., Nosaka N., Kasai M., Aoyama T., Okazaki M., Igarashi O., Kondo K. (2007). Dietary medium- and long-chain triacylglycerols accelerate diet-induced thermogenesis in humans. J. Oleo Sci..

[39] Abe T., Hirasaka K., Kohno S., Tomida C., Haruna M., Uchida T., Ohno A., Oarada M., Teshima-Kondo S., Okumura Y. (2016). Capric acid up-regulates UCP3 expression without PDK4 induction in mouse C2C12 Myotubes. J. Nutr. Sci. Vitaminol..

[40] Shinohara H., Ogawa A., Kasai M., Aoyama T. (2005). Effect of randomly interesterified triacylglycerols containing medium- and long-chain fatty acids on energy expenditure and hepatic fatty acid metabolism in rats. Biosci. Biotechnol. Biochem..

[41] Shinohara H., Wu J., Kasai M., Aoyama T. (2006). Randomly interesterified triacylglycerol containing medium- and long-chain fatty acids stimulates fatty acid metabolism in white adipose tissue of rats. Biosci. Biotechnol. Biochem..

[42] Wen C.P., Wai J.P.M., Tsai M.K., Yang Y.C., Cheng T.Y.D., Lee M.C., Chan H.T., Tsao C.K., Tsai S.P., Wu X. (2011). Minimum amount of physical activity for reduced mortality and extended life expectancy: A prospective cohort study. Lancet.

[43] Lee I.M., Shiroma E.J., Lobelo F., Puska P., Blair S.N., Katzmarzyk P.T., Alkandari J.R., Andersen L.B., Bauman A.E., Brownson R.C. (2012). Effect of physical inactivity on major non-communicable diseases worldwide: An analysis of burden of disease and life expectancy. Lancet.

[44] Bergman B.C., Butterfield G.E., Wolfel E.E., Casazza G.A., Lopaschuk G.D., Brooks G.A. (1999). Evaluation of exercise and training on muscle lipid metabolism. Am. J. Physiol. Endocrinol. Metab..

[45] Amaro-Gahete F.J., Acosta F.M., Migueles J.H., Ponce González J.G., Ruiz J.R. (2020). Association of sedentary and physical activity time with maximal fat oxidation during exercise in sedentary adults. Scand. J. Med. Sci. Sports.

[46] Ooyama K., Wu J., Nosaka N., Aoyama T., Kasai M. (2008). Combined intervention of medium-chain triacylglycerol diet and exercise reduces body fat mass and enhances energy expenditure in rats. J. Nutr. Sci. Vitaminol..

